# The Blood

**Published:** 1874-10

**Authors:** 


### THE BLOOD.

If a cup is filled with small shot, held up and tilted over, they pour out as water, and so with the sands in an hour-glass. The blood is composed of globules, little globes, like shot or sand; all liquids are similarly composed, the thinner they are the smaller are the globules, so small that they cannot be distinguished with the naked eye, but seem to be one mass. The globules of fresh blood are so small, that if laid side by side in a straight line, it takes three thousand of them to make an inch in length. Unhealthy blood, "bad blood," is thick. The spectroscope makes visible one half of a healthy blood globule, hence the time may come when the character of a disease will be indicated by this instrument.

